# High-Density 1D Ionic Wire Arrays for Osmotic Energy Conversion

**DOI:** 10.1007/s40820-025-01976-x

**Published:** 2026-01-01

**Authors:** Jinlin Hao, Cuncai Lin, Min Zhao, Yilin Wang, Xingteng Ma, Lilong Gao, Xin Sui, Longcheng Gao, Kunyan Sui, Lei Jiang

**Affiliations:** 1 Key Laboratory of Marine Bio-Based Fibers of Shandong Province, Qingdao Application Technology Innovation Center of Advanced Fibers and Composites, College of Materials Science and Engineering, Qingdao University, Qingdao, 266071 People’s Republic of China; 2 Laboratory of Bio-Inspired Smart Interfacial Science and Technology of Ministry of Education, School of Chemistry, Beihang University, Beijing, 100191 People’s Republic of China; 3 Key Laboratory of Bio-Inspired Materials and Interfacial Science, Technical Institute of Physics and Chemistry, Chinese Academy of Sciences, Beijing, 100190 People’s Republic of China

**Keywords:** One-Dimensional ionic wire, Self-assembly, High-density ion channels, Ultrahigh ion-exchange capacity, Anti-swelling

## Abstract

Osmotic energy, existing between the seawater and river water, is a renewable energy source, which can be directly converted into electricity by ion-exchange membranes (IEM). In traditional IEMs, the ion transport channels are formed by nanophase separation of hydrophilic ion carriers and hydrophobic segments. It is difficult to realize high-density ion channels with controlled spatial arrangement and length scale of ion carriers. Herein, we construct high-density 1D ion wires as transmission channels. Through molecular design, hydrophilic imidazole groups and hydrophobic alkyl tails were introduced into the repeat units, which self-assembled into 1D ion transporting core and protecting shell along the main chains. The areal density of the ionic wire arrays is up to ~ 10^12^ cm^−2^, which is the highest value. The ionic wires ensure both high ion flux transport and high selectivity, achieving an ultrahigh-power density of 40.5 W m^−2^ at a 500-fold salinity gradient. Besides, the ionic wire array membrane is well recyclable and antibacterial. The ionic wires provide novel concept for next generation of high-performance membranes.

**Supplementary Information:**

The online version contains supplementary material available at 10.1007/s40820-025-01976-x.

## Highlights

- Ultrahigh-Density 1D Ionic Wire Arrays. A high density (~10^12^ cm^−2^) of 1D ionic channels is achieved via self-assembly of a homopolymer, enabling simultaneous high ion selectivity and conductivity for efficient osmotic energy conversion.
- Anti-Swelling Membrane with Superior Performance. The membrane exhibits an ultrahigh ion-exchange capacity (~2.69 meq g^−1^) yet minimal swelling (<10%) due to hydrophobic alkyl shell protection, leading to a breakthrough power density of 40.5 W m⁻² under a 500-fold salinity gradient.
- Multifunctional Design with Antibacterial Properties. The imidazole-functionalized membrane not only enhances osmotic energy harvesting but also provides excellent antibacterial performance, offering a novel strategy for advanced separation membranes.

**Supplementary Information:**

The online version contains supplementary material available at 10.1007/s40820-025-01976-x.

## Graphical abstract

**Figure:**
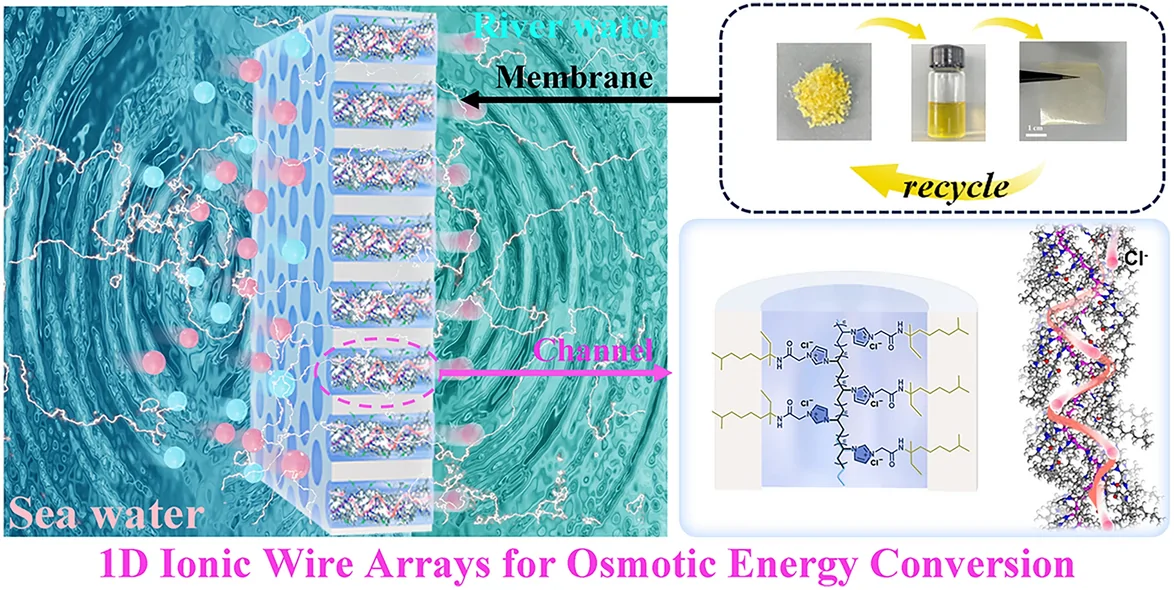

## Introduction

Osmotic energy, existing between the seawater and river water, is a renewable energy source, which can be directly converted into electricity by ion-exchange membranes (IEM) in a reverse electrodialysis system [1–3]. For the ideal IEMs, both high perm-selectivity and conductivity are two key factors, which are influenced by the type, content, and alignment of charged groups [4]. In general, the charged groups need to form interconnected ion channels, which repel co-ions and attract counter-ions through electrostatic interactions [5–7]. In the traditional IEMs, 3D ion-cluster network is self-organized by nanophase separation of hydrophilic ion carriers and hydrophobic segments [7, 8]. However, the spatial arrangement and length scale of ion carriers are often ill-controlled, resulting in a trade-off between ion selectivity and conductivity and limiting the ultimate energy conversion ability [9–13].

Many efforts attend to fabricate advanced IEMs with well-defined nanochannels [14–16]. Among them, the framework materials, including metal–organic frameworks (MOFs) and covalent–organic frameworks (COFs), contain high-density continuous 3D sub-nanochannels [17, 18]. The surface area density can be reached as high as ~ 10^12^ cm^−2^. As a result, these materials exhibit excellent ion regulation ability and ultrahigh-energy conversion ability [19, 20]. Nevertheless, their upscaled membrane fabrication is problematic [21]. In the more practical polymeric systems, ionic block copolymers could self-assemble into nanochannels with long-range order, where ionic groups are nanoconfined with a high local ion concentration [22, 23]. This advantage is beneficial for ion-selective transport. However, considering the tendency of nanophase separation, the width and periodical size of ion nanochannels are mainly dependent on the corresponding segment lengths. The area density is always below ~ 10^11^ cm^−2^ [24, 25]. Further increasing the density of ion nanochannel and maintaining the nanochannel connectivity remain a challenge.

**Fig. 1:**
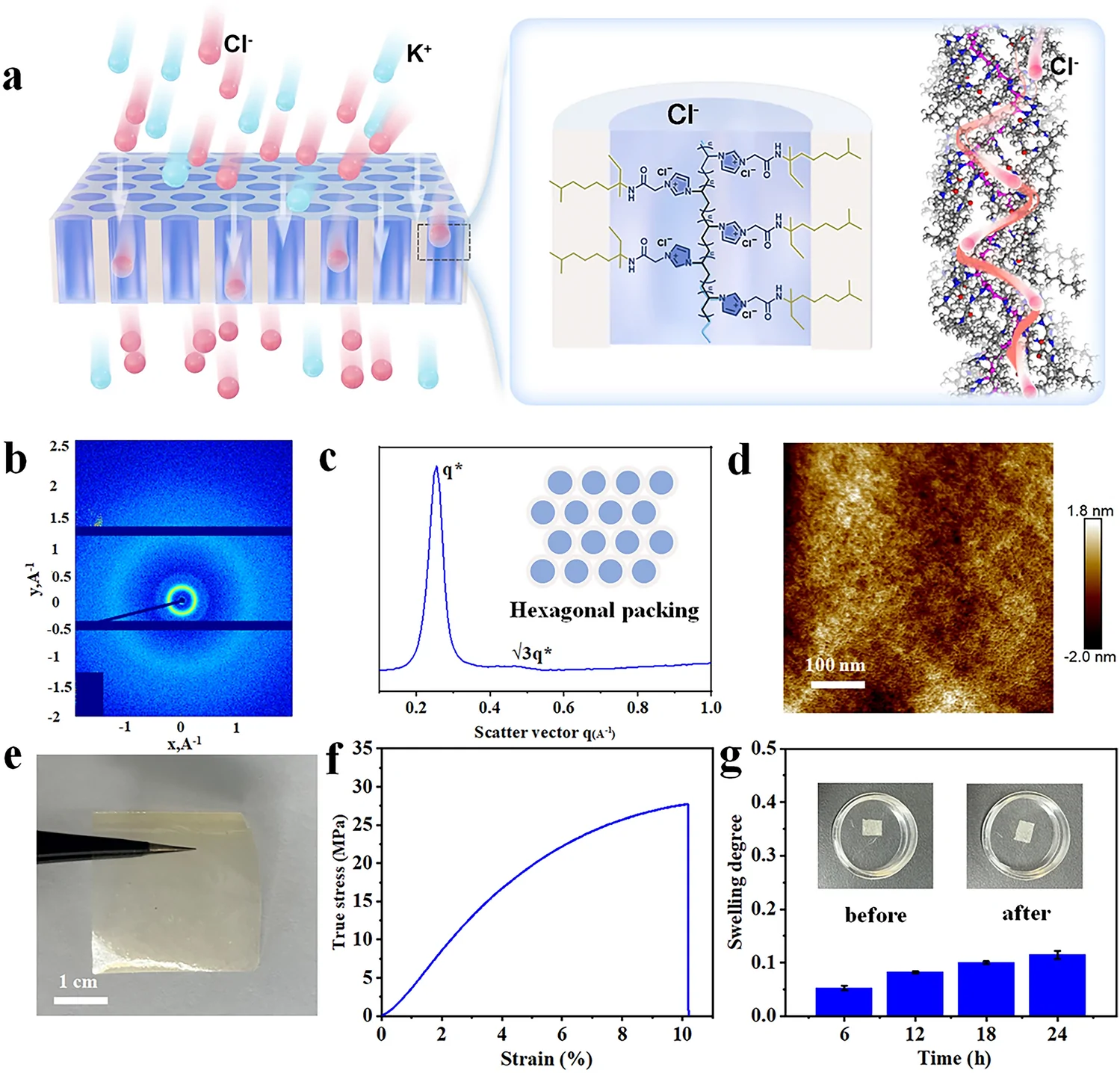
High-density 1D ionic wire membrane. **a** Schematic illustration of the ionic wire array membrane. **b** Two-dimensional WAXD pattern of ionic wire membrane and **c** its 1D intensity profiles. The higher diffraction peak shows a √3 ratio upon the primary peak, indicating a hexagonal packing style. **d** AFM image of the ionic wire membrane, showing high-density channels. **e** Optical photograph of the ionic wire membrane, showing that the membrane can be made in large scale. **f** Stress–strain curve of the ionic wire membrane, showing good mechanical strength. **g** Swelling degree as a function of time, indicating excellent anti-swelling stability, the inset are the images before and after swelling

Here, we demonstrate high-density ion channels formed with 1D ionic wires, exhibiting ultrahigh-power density of osmotic energy conversion. One-dimensional ionic wires are polymerized from an amphiphilic vinyl monomer (1-acetyl tetrahydrolinalool 3-vinylimidazole chloride), with hydrophilic imidazole and long hydrophobic alkyl tail. In the polymer, the long side chains locate as the shell, while imidazole as the core, forming 1D core–shell structure. The side chains prevent the inner imidazole groups from aggregating ion clusters (Fig. 1a). High-density 1D ionic wires (~ 10^12^ cm^−2^) are realized, which have good Cl^−^ selectivity and conductivity. The membrane exhibits ultrahigh-power density of 40.5 W m^−2^ under a 500-fold salinity gradient (5–0.01 M KCl), which is the highest values among the upscaled IEMs. Moreover, the membrane shows excellent antibacterial property because of the imidazole pendant groups. The 1D ionic wires design concept provides a promising approach for osmotic energy conversion and other membrane-based separation processes.

## Experimental Section

### Materials

Tetrahydrolinalool (THL) and concentrated sulfuric acid were purchased from Sigma-Aldrich. Sodium hydroxide, hexaammineruthenium(III) chloride, and AIBN were purchased from TCI. Chloroacetonitrile was purchased from Aladdin. Potassium ferrocyanide trihydrate (99%) was purchased from Innochem. The Rhodamine 6G and the fluorescein sodium salt were purchased from Energy Chemical. Deionized water was prepared from UPT-I-20 T purchased from Sichuan Youpu Ultrapure Technology Co., Ltd. DMF, NaCl, KCl, LiCl, CaCl_2_, and MgCl_2_ were all analytical grade and purchased from Sinopharm Chemical Reagent, China. Seawater from the Yellow Sea near Qingdao. The river water mentioned in the experiment was taken from Qingdao city tap water.

### Synthesis of PTHLCAM-VI

The biomass-based poly(ionic liquid) was synthesized by Ritter reaction, quaternization reaction, and radical polymerization in sequence. Firstly, tetrahydrolinalool (THL, 15.8 g, 0.1 mol) and chloroacetonitrile (9.0 g, 0.12 mol) were added into 100 mL round-bottomed flask, which was immersed in a 0–5 °C ice bath. Concentrated sulfuric acid (11.76 g, 0.12 mol) was diluted to 80% and added drop by drop into the above mixture under vigorously stirring within 30 min (Caution! Concentrated sulfuric acid must be added drop by drop to avoid explosion). After concentrated sulfuric acid was added, the ice bath was removed, and the reaction was raised to 40 °C for 12 h. Sodium hydroxide (9.6 g, 0.24 mol) was dissolved in 200 mL deionized water and drop by drop added into the above reaction mixture under vigorously stirring. The crude product was dissolved in 100 mL of DCM and washed three times with saline solution and dried by magnesium sulfate anhydrous. Finally, the organic layer was concentrated, and the resulting product (named as THLCAM) was dried at room temperature under vacuum for 24 h (yield 93%, almost colorless clear oily liquid at room temperature). Secondly, the above product THLCAM (11.7 g, 0.05 mol) and 1-vinyl imidazole (VI, 7.05 g, 0.075 mol) were added into 250 mL round-bottomed flask with 50 mL DMF, the quaternization reaction was conducted at 80 °C for 12 h. One hundred and fifty mL tert-butyl methyl ether was added to the above reaction mixture under vigorously stirring to remove the unreacted THLCAM and VI. The obtained precipitate was washed twice with tert-butyl methyl ether and dried at room temperature under vacuum for 24 h. The product was named as THLCAM-VI. Thirdly, THLCAM-VI (16.4 g, 0.05 mol) and AIBN (40 mg, 0.25 mmol) were dissolved in 100 mL DMF. Nitrogen was bubbled into the above solution for 30 min to remove dissolved oxygen, then place it in an oil bath preheated to 65 °C for 24 h. After radical polymerization finished, DMF was evaporated at 80 °C. Two hundred mL tert-butyl methyl ether was added into the concentrated solution, the precipitate was washed twice and dried at room temperature under vacuum for 24 h. The obtained pol(ionic liquid) was named as PTHLCAM-VI.

### Preparation of M-PTHLCAM-VI

One g PTHL powder was added in a glass container, add 10 mL of ethanol, and stir until completely dissolved to obtain a PTHLCAM-VI ethanol solution of 100 mg mL^−1^. Take 2 mL of PTHLCAM-VI ethanol solution with a pipette gun and spread it evenly and flatly on a PTFE mold at a constant temperature of 45 °C. Ethanol was allowed to evaporate completely to obtain M-PTHLCAM-VI.

### Characterizations of the Polymer Membrane

The morphology of the polymer membranes was observed by a JSM-7800F scanning electron microscope (JEOL, Tokyo). The interlamellar spacing of membranes was determined by X-ray diffraction tests through Rigaku SmartLab SE. The zeta potentials were measured using Zetasizer Nano ZSE (Malvern PANalytical). The Nyquist plot properties of polymer membranes were investigated using the electrochemical workstation (Shanghai Chenhua CHI760E). A fluorescence spectrophotometer was used to measure the ionic permeability of fluorescein sodium fluorescent dyes and rhodamine 6G. Confocal laser scanning microscopy (ZEISS LSM 900&Axio imager M2) was used to verify the positive and negative ion selectivity of M-TPHL. The mechanical strength of the polymer membrane was measured in the tensile mode by a Shimadzu AGS-X Tester at a loading rate of 0.1 mm min^−1^.

### Ion Transport Regulation Characterization

Current–voltage (I-V) curves were performed with a Keithley 6487 semiconductor microammeter. M-PTHLCAM-VI membrane was mounted in a two-chamber cell. The same concentration of KCl solution was slowly injected into each cell. The current was recorded using a parametric system with a pair of Ag/AgCl electrodes and a recording voltage of 0.1 V (voltage from − 2 ~ 2 V).

### Energy Conversion Characterization

The energy conversion was tested with current recording mode without external power source. The M-PTHLCAM-VI membrane was supported by a polyimide membrane with a circular hole, whose diameter was 200 μm. The corresponding current was recorded with various external resistances. The output power densities were calculated by *P* = *I*^2^*R*/*S*. Where I is the generated current, R is the external resistance, and S is the transportation area, which is the apparent aperture area of the supporting membrane (3.14 × 10^4^ µm^2^).

## Results and Discussion

### Construction of 1D Ionic Wire Array Membrane

The 1D ionic wires are polymeric core–shell structure. The core is the charged imidazole groups, which are connected with the main chains and form 1D ion transport pathways along the main chains. Outside the imidazole core, long amorphous alkyl groups are grafted, of which the carbon number and the architecture are key parameters. Due to the steric effect of the alkyl groups, hydrophobic shells are formed surrounded the 1D imidazole cores, preventing from the solvation effect [26]. The molecular simulation shows the 1D core–shell structure (Fig. 1a). The successful monomer synthesis and free radical polymerization are carried on (Figs. S1-S6). The resultant polymer aggregates into cylindrical structure. From the wide-angle X-ray scattering (Fig. 1b, c), we can see a primary scattering peak centered around *q* value (0.25 nm^−1^), corresponding to a d-spacing of ~ 2.47 nm. Higher order halo with a √3 ratio over the primary peak indicates a hexagonal packing, which is confirmed by the AFM image (Fig. 1d). The observed nanophase separation originates from hydrophobic/hydrophilic interactions among side chains, though the backbone structure restricts long-range ordering, resulting in only short-range molecular organization. On the surface, we can see nano-dots, the cross-section of the polymer, with an average distance of ~ 3.0 nm (Fig. S7). The areal density of ionic wires is calculated to be ~ 10^12^ cm^−2^. It is worth noting that the density is highest among the IEMs. The polymer has good solubility in organic solvents such as ethanol, which allows upscaled membrane fabrication (Fig. 1e) [24, 27, 28]. The obtained membrane exhibits good mechanical strength (~ 25 MPa, Fig. 1f). Meanwhile, owing to the long alkyl protection, the membrane is not soluble in water. Thus, the membrane shows excellent anti-swelling stability (Fig. 1g). Compared to the traditional IEMs, our membrane has an ultrahigh ion-exchange capacity (IEC) value of up to ~ 2.69 meq g^−1^ and shows a low water uptake of ~ 10%. This feature benefits for the ion-selective transport in aqueous condition.

### Transmembrane Anion Transport

**Fig. 2:**
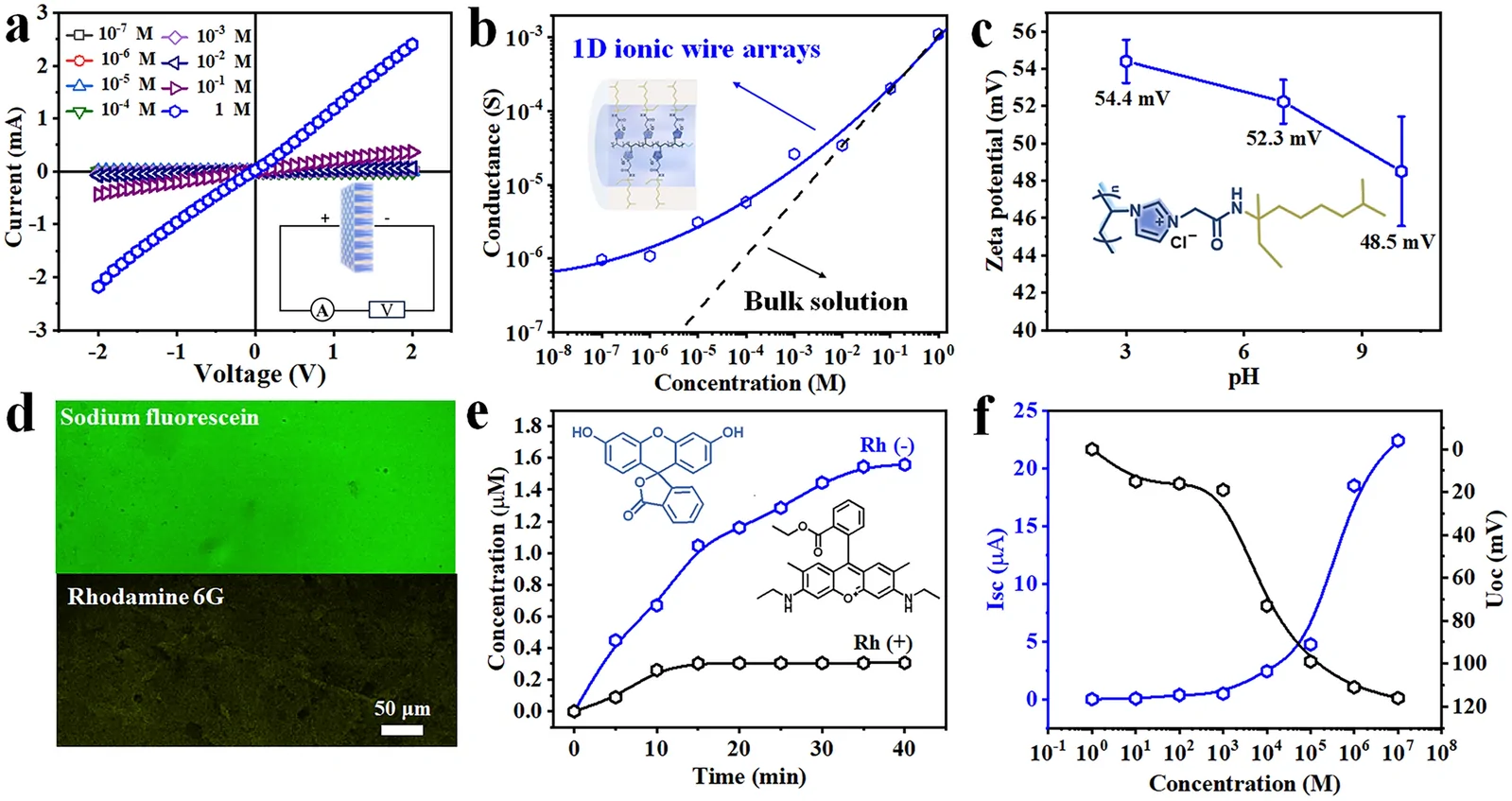
Anion-selective transport. **a** I-V curves of the ionic wire membrane under a range of KCl solution concentrations. **b** Conductance measurement in KCl electrolyte. The ionic conductance deviates from bulk value (blue line) at low concentration region, indicating a charge-governed ion transport. **c** Zeta potential of the ionic wire membrane at different pH values. **d** Fluorescence images of the membrane dyed by positively charged fluorescent dye (Rhodamine 6G, Rh ( +)) and negatively charged fluorescent dye (Sodium fluorescein, Rh (−)). **e** Time–concentration curves of two oppositely charged fluorescent dye permeation experiments. Negative charged dyes can be transported across membranes in large quantities, while positive charged dyes are rarely transported. **f** Variations of *U*_oc_ and *I*_sc_ as a function of KCl concentration gradient

The ionic wire array membrane shows excellent ionic transport ability. The ion transport across the membrane was tested by a two-compartment electrochemical cell (Figs. S8 and S9). A series of I-V curves were recorded with concentrations from 10^−7^ to 1 M (Fig. 2a) [29]. The curves are all linear, and the conductance values are obtained. As shown in Fig. 2b, the conductance obeys the bulk rule with a linear tendency in the high concentration region (> 10^−2^ M) and departs from the bulk value and gradually approaches a plateau in the low concentration region. In low concentration region, the thickness of electric double layer increases and becomes comparable to the channel dimension. Due to the positive surface charge carried by ionic wire membrane, anions will accumulate in the channels. Under this condition, the ion concentration inside the channel is determined by ionic wire membrane rather than the bulk concentration. Thus, the ion conductance in low concentration region will increase. The charge-regulated ionic transport property is determined by introduction of charge carriers in membrane [30, 31]. The existence of positive charges on the membrane surface was confirmed by the characterization of zeta potential. As shown in Fig. 2c, the membrane has positive potential over a wide range of pH. The surface potential values of ionic wire membrane at pH 3, 7, and 10 were tested using zeta potential and were 54.4, 52.3, and 48.5 mV, respectively (Fig. 2c). The surface charges benefit for ion adsorption.

The ionic wire membrane with high-density, positively charged imidazole displays excellent anion selectivity. Firstly, we used two fluorescent dyes (sodium fluorescein and rhodamine 6G) with opposite charges to probe the selectivity (Fig. S10) [32]. After contacting with the negatively charged dye, strong fluorescent emission can be seen by confocal laser scanning microscopy. In contrast, there is nearly no fluorescent emission after contacting with rhodamine 6G (Fig. 2d). It means that negative ions are easy to be adsorbed by the membrane. Furthermore, under the dye concentration gradients, sodium fluorescein diffuses across the membrane with much higher rate than that of rhodamine 6G (Fig. 2e). We also used cyclic voltammetry (CV) tests to verify the ion selectivity in the presence of electroactive redox probes, [Fe(CN)_6_]^3−^ and [Ru(NH_3_)_6_]^3+^ (Fig. S11). The CV curve obtained with [Fe(CN)_6_]^3−^ shows a higher electrochemical response, meaning that anions selectively permeate through ionic wire membrane toward the electrode [33].

The ion selectivity can also be quantitatively researched by I-V measurements under asymmetric KCl solutions. Open-circuit voltages (*U*_oc_) are obtained under different concentration gradients (Figs. 2f and S12). *U*_oc_ can be expressed by the following Eq. (1) [34]:

1 $$U_{{{\mathrm{OC}}}} = \frac{{RT}}{F}\ln \left( {\frac{{C_{{{\mathrm{high}}}} }}{{C_{{{\mathrm{low}}}} }}} \right)\left( {t_{ + } - t_{ - } } \right)$$

where *t*_+_ and *t*_*-*_ are the ion transference numbers for K^+^ and Cl^−^, respectively. *R*, *T,* and *F* are the universal gas constant, the absolute temperature, and the Faraday constant, respectively. *C*_high_ and *C*_low_ are KCl solution concentration values for the high and low concentration sides, respectively. The Cl^−^/K^+^ selectivity ratio (*S*) can be obtained by Eq. (2),

2 $$S = \frac{{{\mathrm{t}}_{ - } }}{{1 - t_{ - } }}$$

The *S*-value is calculated based on the data presented in Fig. S12. At tenfold concentration gradient, the *S* value reaches as high as ~ 0.99, indicating that the ionic wire membrane shows outstanding anion selectivity.

### Osmotic Energy Conversion

**Fig. 3:**
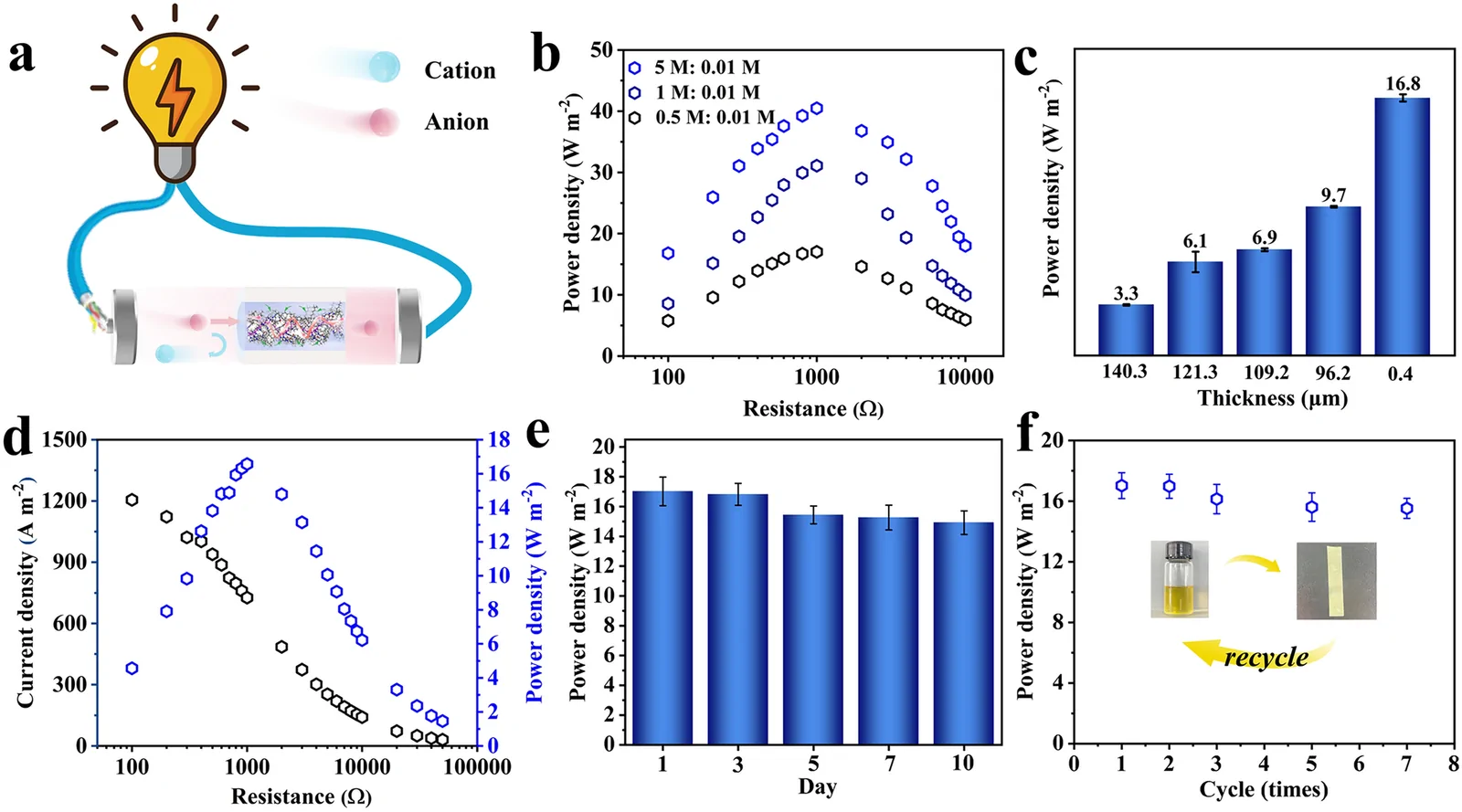
Osmotic energy conversion. **a** Schematic diagram of the osmotic energy harvesting system based on the ionic wire membrane. **b** Power densities of the ionic wire membrane under different concentration gradients. **c** Output power density of the ionic wire membrane with different thicknesses. **d** Output power density of the ionic wire membrane under seawater and river water. **e** Stability of the ionic wire membrane-based energy conversion device under 50-fold concentration gradient conditions. **f** Stability of the ionic wire membrane under multiple cycles for osmotic energy conversion

The high-density 1D ionic wire arrays with excellent ion selectivity and conductivity exhibit ultrahigh osmotic energy conversion capability. The electrical energy obtained by the external circuit of ionic wire membrane-based osmotic energy conversion device is evaluated by adjusting the resistance of the resistor in the device (Fig. 3a). The output power density (*P*) can be obtained by Eq. (3):

3 $$P = I^{2} R/S$$

where *R* is the test resistance value of the adjustable resistor, *I* is the current value under the corresponding test resistance, and *S* is the effective test area of the ionic wire membrane [35]. The concentration of one side of the cell is set as 0.01 M KCl, while the other side concentration is set as 0.5, 1.0, and 5.0 M, with a concentration gradient of 50-, 100-, and 500-fold, respectively. With increasing out resistance, the output power density reaches a maximum value when the resistance is equal to the internal resistance of the membrane. The maximum output power density reached 17.0–40.5 W m^−2^ from 50- to 500-fold concentration gradient, which is excellent value among the upscaled membrane (Fig. 3b). At lower concentration gradient, the power density decreases. The performance of the osmotic energy conversion increases with decreasing membrane thickness (Figs. 3c and S13). We also tested the performance in the natural solutions. By using natural river water and seawater, the power density reaches 16.6 W m^−2^ (Fig. 3d). The ion wire membrane has excellent stability. We evaluated the membrane under different pH values, and the output power densities retain at high level over wide pH range. The performance is highest at low pH condition (17.6 W m^−2^ with pH = 3) and lowest at high pH condition (14.6 W m^−2^ with pH = 10) (Fig. S14). Meanwhile, over a long-time test, the power density remains almost unchanged (Figs. 3e and S15). Generally, higher power density outputs can be achieved within reduced spatial scales (Fig. S16). This phenomenon, which aligns with prior publications [36, 37], stems from internal resistance of the device [21]. In addition, the power density outputs of various anion solutions were systematically researched (Fig. S17). The results indicate a significantly lower performance compared to KCl solution (16.8 W m^−2^), primarily due to the low ion diffusion coefficient and the absorption with surface functional groups. It is worth noting that the ionic wire membrane is totally recyclable. Different other IEMs, our membrane has good solubility in common solvents. Thus, the membrane can be reproduced by a solution casting process. And the power density maintains at a high value (retain 91.7%) (Fig. 3f).

### Antibacterial Property

**Fig. 4:**
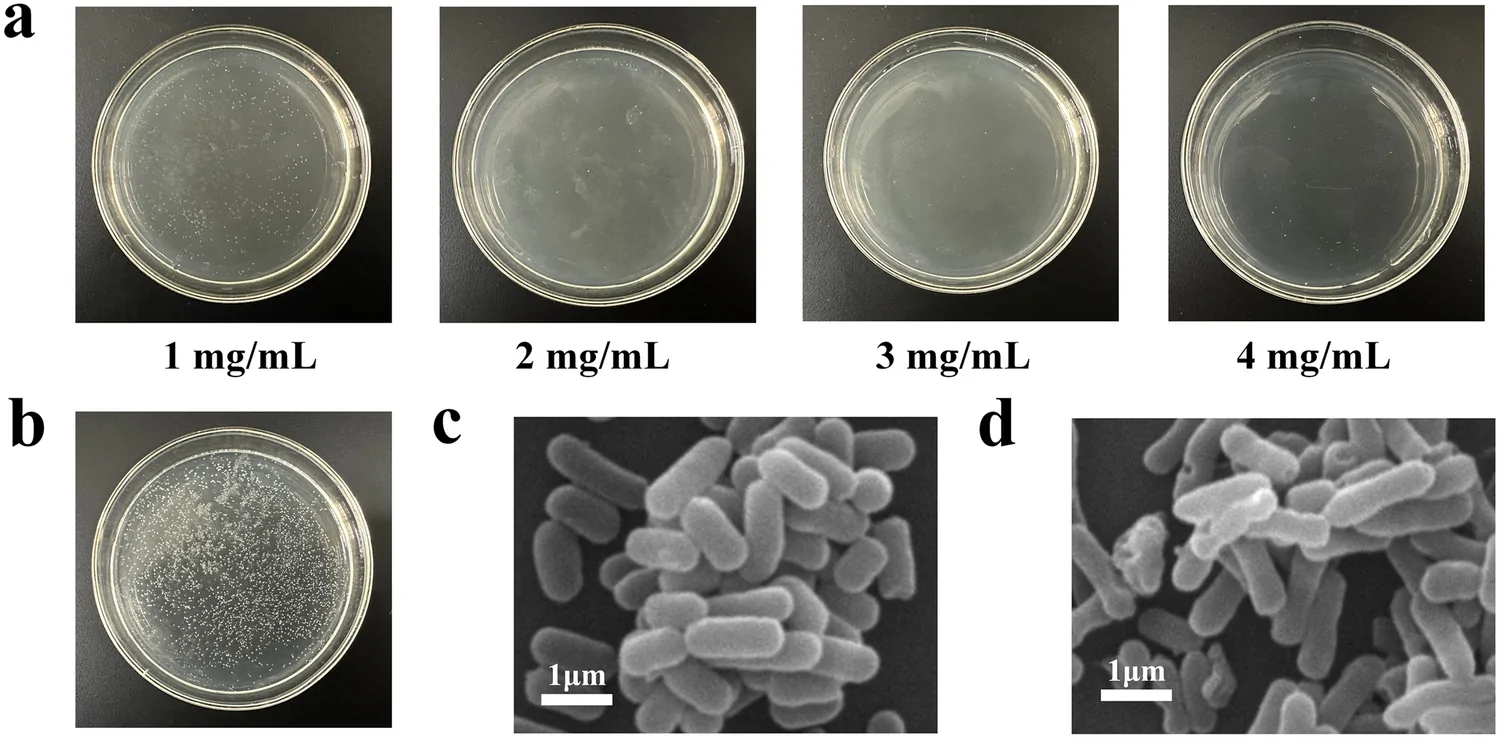
Antibacterial properties. **a** Comparative image of bacterial inhibition by different concentrations of ionic wires membrane. **b** Control group without ionic wires membrane SEM images of the bacteria before **c** and after **d** co-culture with the ionic wire membrane, showing that bacteria shriveled and collapsed

Besides, the ionic wire array membrane shows excellent antibacterial property. In natural marine and riverine environments, the activities of microorganisms have a fatal impact on the membranes [38–40]. The abundant imidazole groups ensure the antibacterial property. To address the antibacterial property, we added the post-grinding membrane into the culture medium with E. coli. The polymer concentration was divided into four groups of 1, 2, 3, and 4 mg mL^−1^, respectively, and the other four groups of culture medium without adding polymer were used as blank control. The activated E. coli solution was added equally to both the experimental and blank groups. After 8 h of cultivation, equal amounts of bacterial liquid from each group were transferred to solid media. Bacterial growth was observed after an additional 8 h of continuous cultivation. As depicted in Fig. 4b, the comparison groups exhibited a substantial bacterial population. Conversely, when incubated with 1 mg mL^−1^ ionic wire, a notably reduced bacterial count was observed in the laboratory group (Fig. 4a). The antibacterial rate was obtained by conducting colony counts on the control group and the experimental group. The experimental group demonstrated antibacterial rates of 88.4%, 97.4%, 98.5%, and 98.9% at concentrations of 1, 2, 3, and 4 mg mL^−1^, respectively. This profound antibacterial effect is attributed to the inherently bacteriocidal nature of the imidazolium salt group, which also enhances hydrophilicity and thus facilitates bacterial contact and elimination. We also use SEM to observe the morphological changes of the bacteria before and after co-culture with the ionic wire membrane. Initially, the bacterial structure was plumpness, and the shape was regular (Fig. 4c). After mixed culturing with ionic wire, the bacteria showed irregular morphological structures such as shriveled and collapsed, which further confirmed the antibacterial effect of the membrane (Fig. 4d).

## Conclusions

Herein, we have successfully demonstrated the fabrication of a high-density one-dimensional ionic wire as ion channels through polymer self-assembly. This innovative design enables the generation of substantial power densities, reaching up to 40.5 W m^−2^ in a remarkable 500-fold salinity gradient. The achievement of ion channels constructed from one-dimensional ionic wires is attributed to our refined molecular design approach. By incorporating hydrophilic ionic and hydrophobic carbon chains into the homopolymer repeating units, we have attained ion channel densities of up to 10^12^ cm^−2^, exhibiting outstanding ionic selectivity and enabling efficient salinity gradient energy conversion. Furthermore, the incorporation of imidazolium salt groups has imparted the membrane with superior antimicrobial properties. Notably, this membrane exhibits excellent recyclability, maintaining stable performance over multiple cycles. From molecular design perspective, the development of polymeric ion-selective membranes with high channel density and surface charge density presents a promising route for industrial-scale applications.

## Supplementary Information

Below is the link to the electronic supplementary material.

Supplementary file1 (DOCX 1483 KB)
