# Supplementary material for: High-Density 1D Ionic Wire Arrays for Osmotic Energy Conversion
Source: Nanomicro Lett. 2026 Jan 1;18:125. doi: 10.1007/s40820-025-01976-x (PMC12756209; doi:10.1007/s40820-025-01976-x)
Supplement: Supplementary file 1 — Supplementary file1 (DOCX 1483 KB) [file 40820_2025_1976_MOESM1_ESM.docx]

Supporting Information for

**High-Density 1D Ionic Wire Arrays for Osmotic Energy Conversion**

Jinlin Hao^1, #^, Cuncai Lin^1, #^, Min Zhao^2^, Yilin Wang^1^, Xingteng Ma^1^, Lilong Gao^1,^ *, Xin Sui^1,^ *, Longcheng Gao^2,^ *, Kunyan Sui^1,^ *, Lei Jiang^3^

^1^ Key Laboratory of Marine Bio-based Fibers of Shandong Province, Qingdao Application Technology Innovation Center of Advanced Fibers and Composites, College of Materials Science and Engineering, Qingdao University, Qingdao 266071, P. R. China

^2^ Laboratory of Bio-inspired Smart Interfacial Science and Technology of Ministry of Education, School of Chemistry, Beihang University, Beijing 100191, P. R. China

^3^ Key Laboratory of Bio-inspired Materials and Interfacial Science, Technical Institute of Physics and Chemistry, Chinese Academy of Sciences, Beijing 100190, P. R. China

^#^ Jinlin Hao and Cuncai Lin contributed equally to this work.

*Corresponding authors. E-mail: [suixin_1991@126.com](mailto:suixin_1991@126.com) (Xin Sui); [gaolilong@qdu.edu.cn](mailto:gaolilong@qdu.edu.cn) (Lilong Gao); [lcgao@buaa.edu.cn](mailto:lcgao@buaa.edu.cn) (Longcheng Gao); [sky@qdu.edu.cn](mailto:sky@qdu.edu.cn) (Kunyan Sui)

**Supplementary Figures**

**Fig. S1** Synthesis route of PTHLCAM-VI





**Fig. S2** ^1^H NMR spectra of THLCAM





**Fig. S3** ^1^H NMR spectra of PTHLCAM-VI


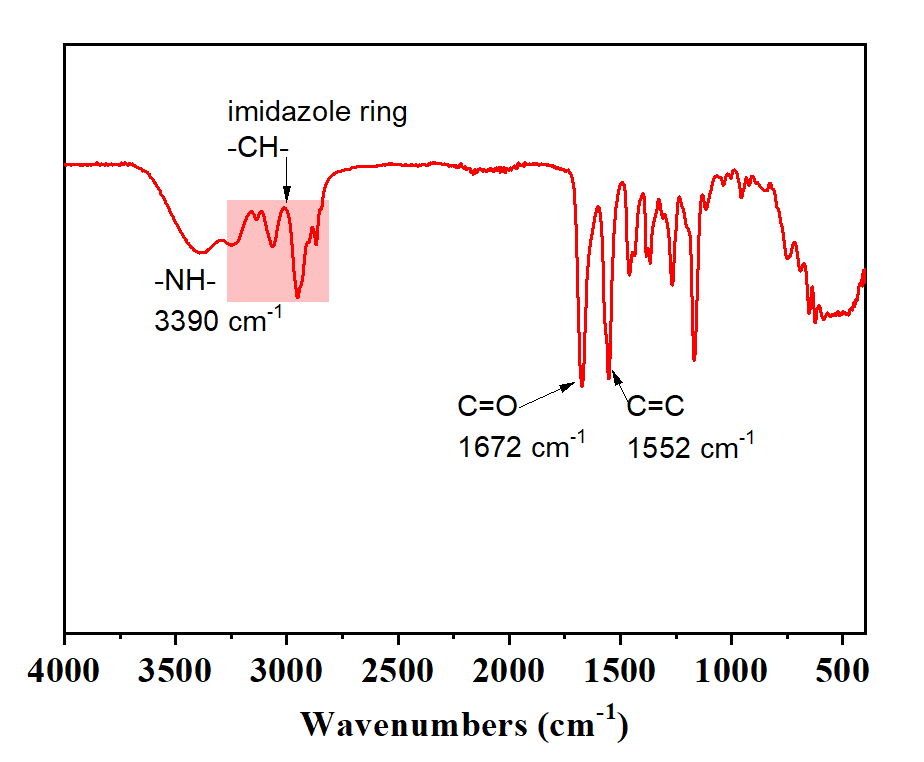


**Fig. S4** IR spectra of THLCAM-VI





**Fig. S5** ^1^H NMR spectra of THLCAM-VI

**
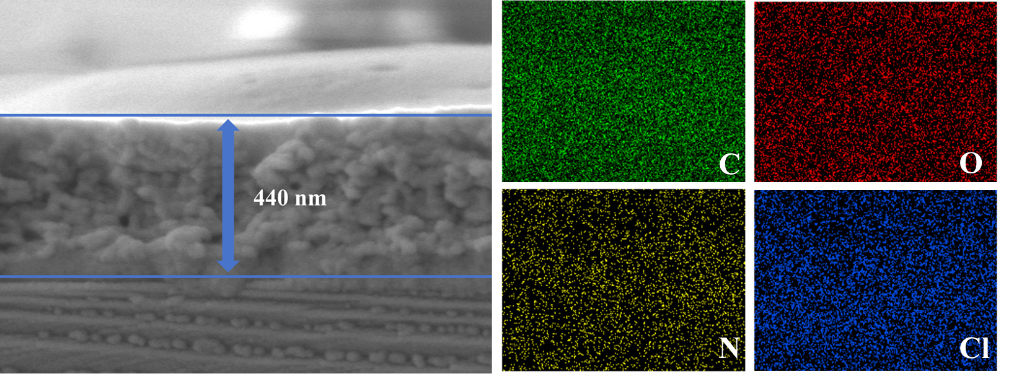
**

**Fig. S6** Cross-sectional SEM image and EDX mapping of the M-PTHLCAM-VI


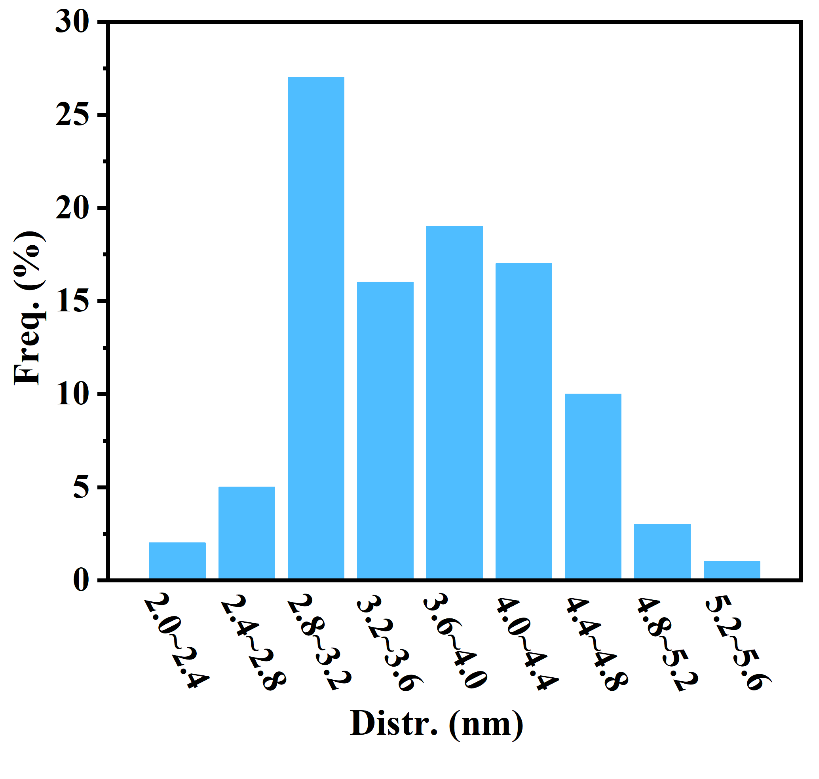


**Fig. S7** AFM test pore size distribution statistics


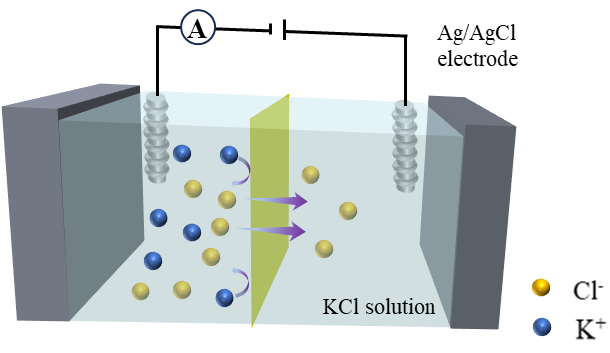


**Fig. S8** Schematic of two-chamber electrochemical cell


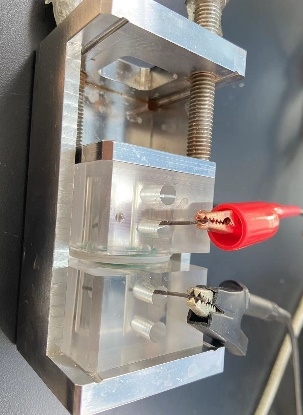


**Fig. S9** Optical image of two-chamber cell

**
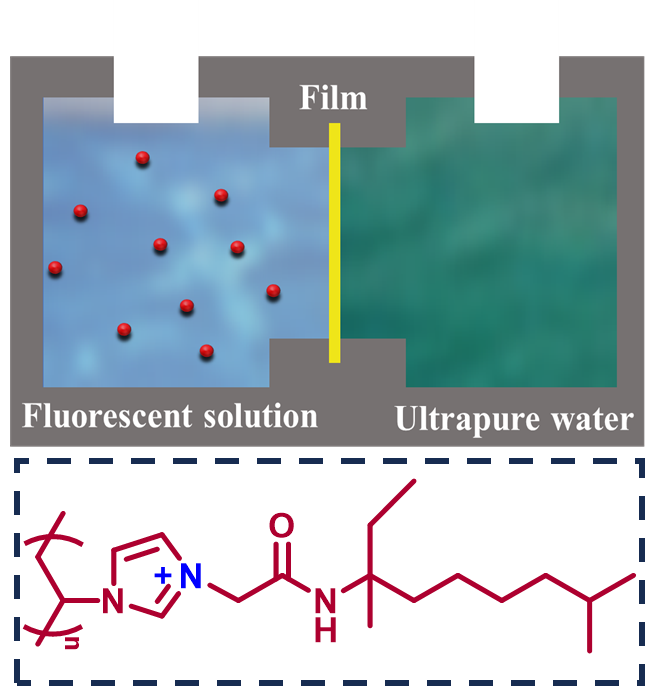
**

**Fig. S10** Image of bespoke two-cell test setup


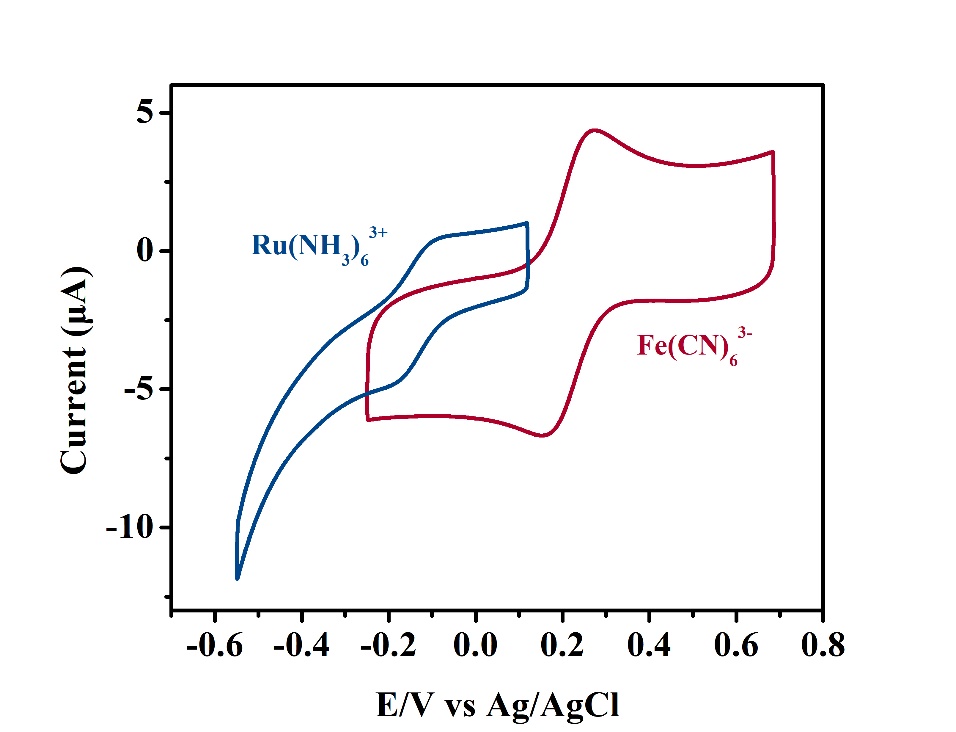


**Fig. S11** CV curves of the PTHLCAM-VI membrane when using [Fe(CN)_6_]^3−^ as the cationic electroactive probe and [Ru(NH3)_6_]^3+^ as the anionic electroactive probe


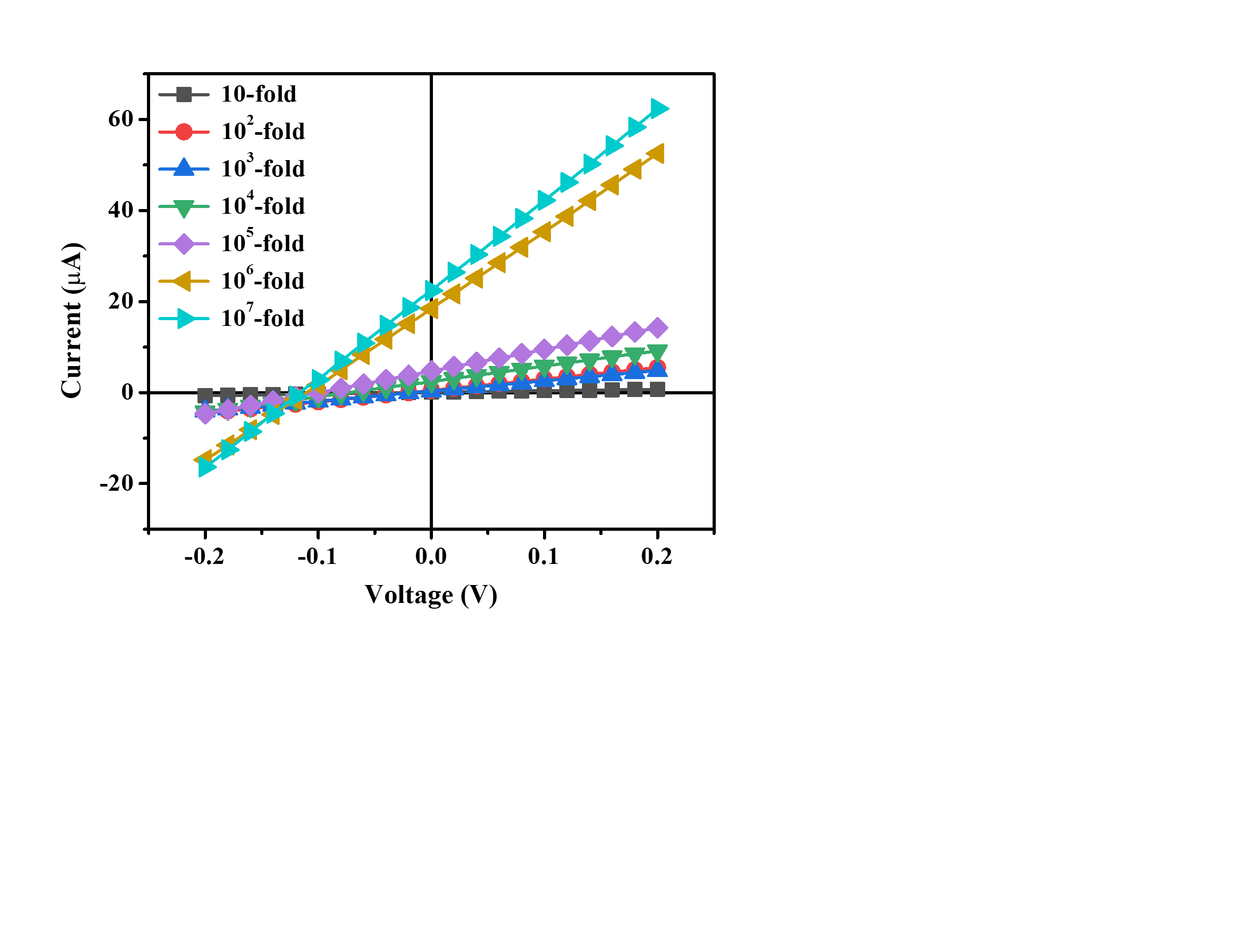


**Fig. S12** I-V curves of the M-PTHLCAM-VI in KCl solutions of different concentration gradients


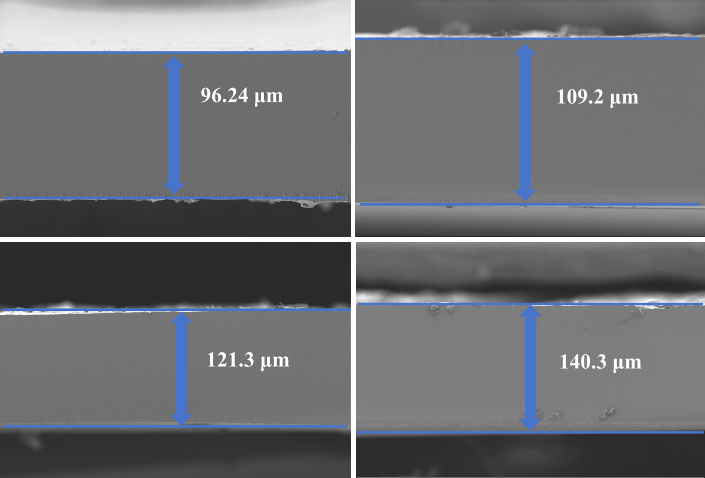


**Fig. S13** SEM of PTHLCAM-VI membranes with different thickness

**Fig. S14** Power densities of the PTHLCAM-VI membrane under different pH conditions


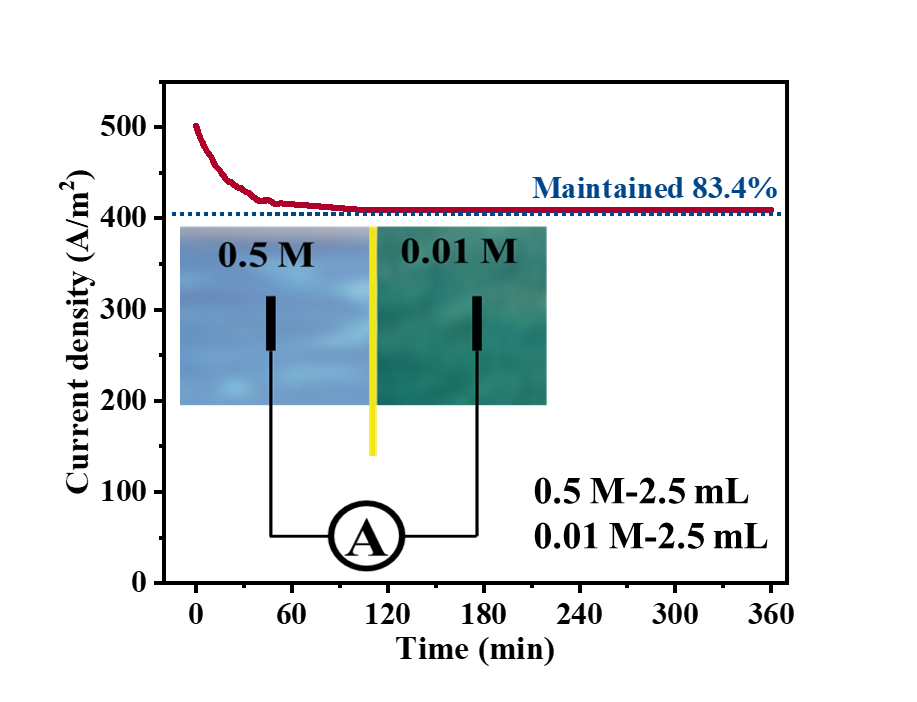


**Fig. S15** Current-time curve of system working with no electrolyte replenishing

**Fig. S16** The relationship between the power densities and testing area of the PTHLCAM-VI membrane under 0.5 M KCl and 0.01 M KCl

**Fig. S17** Power densities of the PTHLCAM-VI membrane in various salt systems
